# Left and Right Ventricle Late Remodeling Following Myocardial Infarction in Rats

**DOI:** 10.1371/journal.pone.0064986

**Published:** 2013-05-31

**Authors:** Ivanita Stefanon, María Valero-Muñoz, Aurélia Araújo Fernandes, Eduardo Hertel Ribeiro, Cristina Rodríguez, Maria Miana, José Martínez-González, Jessica S. Spalenza, Vicente Lahera, Paula F. Vassallo, Victoria Cachofeiro

**Affiliations:** 1 Department of Physiological Sciences, Federal University of Espirito Santo, Vitoria, Espirito Santo, Brazil; 2 Department of Physiology, Universidad Complutense, Madrid, Spain; 3 Centro de Investigación Cardiovascular (CSIC-ICCC), Institut d’Investigació Biomèdica Sant Pau, Barcelona, Spain; Virginia Commonwealth University, United States of America

## Abstract

**Background:**

The mechanisms involved in cardiac remodeling in left (LV) and right ventricles (RV) after myocardial infarction (MI) are still unclear. We assayed factors involved in collagen turnover in both ventricles following MI in rats either presenting signs of heart failure (pulmonary congestion and increased LVEDP) or not (INF-HF or INF, respectively).

**Methods:**

MI was induced in male rats by ligation of the left coronary artery. Four weeks after MI gene expression of collagen I, connective tissue growth factor (CTGF), transforming growth factor β (TGF-β) and lysyl oxidase (LOX), metalloproteinase-2 (MMP2) and tissue inhibitor metalloproteinase-2 (TIMP2) as well as cardiac hemodynamic in both ventricles were evaluated.

**Results:**

Ventricular dilatation, hypertrophy and an increase in interstitial fibrosis and myocyte size were observed in the RV and LV from INF-HF animals, whereas only LV dilatation and fibrosis in RV was present in INF. The LV fibrosis in INF-HF was associated with higher mRNA of collagen I, CTGF, TGF-β and LOX expressions than in INF and SHAM animals, while MMP2/TIMP2 mRNA ratio did not change. RV fibrosis in INF and INF-HF groups was associated with an increase in LOX mRNA and a reduction in MMP2/TIMP2 ratio. CTGF mRNA was increased only in the INF-HF group.

**Conclusions:**

INF and INF-HF animals presented different patterns of remodeling in both ventricles. In the INF-HF group, fibrosis seems to be consequence of collagen production in LV, and by reductions in collagen degradation in RV of both INF and INF-HF animals.

## Introduction

Heart failure (HF) due to myocardial infarction (MI) is one of the major health care issues in the world and leads to a high rate of hospitalization and mortality. MI is accompanied by a wound-repairing process of the damaged area. This process involves a cascade of coordinated events resulting in both replacement of injured contractile tissue by a fibrotic scar and a remodeling in the remaining ventricle [1]–[3].

Even though it is well established that the development of HF depends on the size of the scar area [4], we have recently demonstrated that infarcted rats presenting scar areas between 30–50% of the left ventricle do not always develop typical signs of HF such as pulmonary congestion and increased left ventricle end diastolic pressure. In addition, both groups presented different pattern of vascular reactivity and remodeling process in the non-ischemic myocardium [5], [6].

Ventricular remodeling following MI involves complex biochemical, molecular and morphological alterations in both ischemic and remote non-infarcted myocardial area. This remodeling involves phenotypic changes in the myocytes as well as in the extracellular matrix, which results in myocardial fibrosis consequence of an imbalance between its production and degradation [7]. Collagen synthesis, preferentially mediated by myofibroblasts, is induced in response to different stimuli; these include mechanical stress, vasoactive factors such as angiotensin II and growth factors such as transforming growth factor β (TGF-β), which can act directly or through the up- regulation of connective tissue growth factor (CTGF) [8]–[10]. Collagen degradation is mediated by a family of zinc-containing endoproteinases– matrix metalloproteinases (MMPs). These enzymes are found in the heart at low levels in normal conditions but can be up-regulated after MI in response to inflammatory cytokines and TGF-β. Their activity is modulated by endogenous inhibitors of MMP (TIMPs) which bind MMPs in a stoichiometric relation [11]. Another critical step in collagen fibre synthesis is the cross-linking of fibrillar collagen by the action of lysyl oxidase (LOX), an extracellular enzyme that confers the tensile strength and mechanical properties of collagen fibres [12], [13]. Interestingly, collagen cross-links contribute to increased ventricular stiffness and reduced compliance; these could thus compromise ventricular function in cardiac diseases [14], [15]. Growth factors such as TGF-β and CTGF and proinflammatory cytokines control LOX production in the heart and other tissues [13], [16].

Although several factors involved in ventricular remodeling following MI have been identified, the late mechanism responsible for fibrosis in the non-ischemic myocardium of left and right ventricles that can trigger the development of functional alterations is not yet well understood. Moreover, whether these changes are associated with functional alteration in both ventricles is not well-established. Therefore, the aim of this study was to evaluate the different factors involved in the interstitial collagen turnover late after MI in animals presenting signs of HF or not, and whether these changes could account for the functional and morphological alterations in the non-ischemic myocardium of left and right ventricles in a rat model.

## Methods

### Experimental Design and Animals

Male Wistar rats (220–240 g) were obtained from colonies maintained at Federal University of Espirito Santo. Rats were housed at constant room temperature (20 to 22°C), humidity (50 to 60%) and light cycle (12∶12 h light-dark), with free access to standard rat chow and tap water. The study conforms to the *Guide for the Care and Use of Laboratory Animals* published by the US National Institutes of Health (NIH Publication No. 85-23, revised 1996) and follows the guidelines of the *Committee on Care and Use of Laboratory Animal Resources* of the University of Espirito Santo, Vitória, Espirito Santo. MI was induced by ligation of the left coronary artery as previously described [5], [17]. Briefly, the rats were anesthetized using Ketamine (50 mg/kg; *i.m.*) and Xylazine (5 mg/kg; *i.m*.) and underwent left lateral thoracotomy between the fourth and the fifth intercostal space. After exteriorization of the heart, the left atrium was pushed aside and the left coronary artery was ligated with 6.0 mononylon thread (Shallon, needle: 2 mm) between the exit point of the pulmonary artery and left atrium. Next, the heart was returned to the thorax and it was closed with 1.0 cotton sutures. Weight-matched rats were used as controls and underwent all surgical steps except for coronary ligation (SHAM group). After recovery, the animals were kept in collective cages with free access to food and water.

### Hemodynamic Measurement

MI rats were anesthetized with urethane (1.2 g/kg; *i.p*.) four weeks later. The right jugular vein and right carotid artery were carefully isolated to avoid damage to any surrounding nerves. A taper-ending polyethylene cannula (PE 50) filled with heparinized saline (100 U/ml) was inserted into the right carotid artery for measurements of systolic arterial pressure (SAP), diastolic arterial pressure (DAP), heart rate (HR) and then inserted into the left ventricle for measurements of left ventricular systolic pressure (LVSP), left ventricle end diastolic pressure (LVEDP) and positive (+dP/dt) and negative (−dP/dt) rates of pressure development. Another catheter was inserted into the right ventricle through the right jugular vein in order to measure the right ventricular systolic pressure (RVSP), right ventricle end diastolic pressure (RVEDP) and +dP/dt and −dP/dt rates of pressure development. Arterial and ventricular pressures were determined by connecting the catheter to a pressure transducer (TSD104A) coupled to a MP100 amplifier and to an acquisition system (MP 100 Biopac Systems, Inc., CA, USA) for real-time pressure and HR recording and for subsequent analysis. The animals were killed and the lung and both right and left ventricles were separated and weighed. HF was considered to be present when at least two of the following criteria were met: Lung/BW ≥ Lung/BW _sham_ +2 SD (Lung wet weight; BW = body weight; SD = standard deviation), RV/BW ≥ RV/BW _sham_ +2 SD (Right ventricle wet weight; BW = body weight; SD = standard deviation) and LVEDP greater than 15 mmHg [5], [6], and a concomitant reduction in the indices of LV contractility and relaxation (INF-HF group). The animals that did not fulfil these criteria were included in the INF group.

### Scar Area Quantification

Scar area was determined as previously reported [18]. Briefly, heart was cut into three different transversal sections (apex, middle ring and base). From the middle ring, 5 µm sections were cut at 100 µm intervals and stained with picrosirius red staining and assessed by light microscopy. The scar size (fraction of the infarcted left ventricle) was calculated as the average of all middle ring slices and expressed as percentage of left ventricle, which included the septum length. Rats with an extensive scar size between 40 to 60% were included in the study.

### Cardiac Interstitial Collagen Quantification

Tissue samples from middle ring of left and right ventricles were dehydrated, embedded in paraffin and cut into sections of 5 µm thickness. These sections were stained with picrosirius red staining (Aldrich Chemical Company). Interstitial collagen quantifications in right and left ventricles were performed using an image analysis system (BEL Engineering, Top Light B2, Italy). The area of interstitial fibrosis was identified after excluding the vessel area and scar area from the region of interest and determined as the ratio of interstitial collagen deposit to the total remaining tissue area from each ventricle. For each sample, 10 to 15 fields were analyzed with a 40 X objective lens under transmitted light.

### Morphometric Analysis

Hearts were arrested in diastole using urethane before harvesting, and then dehydrated, embedded in paraffin for morphometric analyses. Myocyte cross-sectional area was used for the evaluation of the degree of ventricular hypertrophy [19]. The blocks made in this manner were cut with a microtome to obtain 5 µm thick sections (5–7 for each heart), which were placed onto glass slides and stained according to the hematoxylin-eosin technique using an image analysis system from BEL Enginteering, Top Light B2, Italy. For each section, 10–15 fields were analysed with a 40 X objective lens under transmitted light. A total of at least 60–70 cells were measured by group. The cross sectional area of both left and right ventricle cavities, free wall thickness and diameters of both ventricles as well as septum thickness were also measured. Two sections for each animal at the midregion area were analysed with a 1.5X objective lens under transmitted light.

### Gene Expression

A sample from the survival left ventricle and right ventricle tissue were used to RNA extraction. Total RNA extraction from left and right ventricles was performed using a nucleic acid purification lysis solution (RNeasy™, Qiagen). One µg of total RNA was taken to perform Reverse Transcription. Genomic DNA was eliminated with a mixture of gDNA Wipeout Buffer and RNase free water incubated for 2 min at 42°C. Then, the mixture was completed with Quantiscript Reverse Transcriptase, Quantiscript RT Buffer and RT Primer Mix. This mixture was incubated for 15 min at 42°C and 3 min at 95°C (Qiagen, Sciences, Maryland, USA) [20].

Gene expression was quantified by real-time PCR using TaqMan™ primers and probes for Collagen I, TGF-β, CTGF, MMP-2, TIMP-2, 18S ribosomal (Roche) and LOX (Applied Biosystems) (Table 1). Real-time PCR was performed using a fluorescence temperature cycler (Smart Cycler; Cepheid, Sunnyvale, California, USA). The 2^−ΔΔCT^ method analyzes relative changes in gene expression from real-time quantitative PCR experiments [21]. Data were normalised by 18S ribosomal levels and expressed as % relative to controls (SHAM).

**Table 1:** Primers and probes used in quantitative real-time PCR Analysis.

GENE	PRIMERS AND PROBES
	Sense 5′ GGCTACCACATCCAAGGAAG 3′
18S	Antisense 5′ CAATTACAGGGCCTCGAAAGA 3′
	Probe 5′ TEX-CGCAAATTACCCACTCCCGACCC-BBQ 3′
	Sense 5′ CCCTGCAGCTGGAGAGTGT 3′
IL-1β	Antisense 5′ TGGTCTTGACTTCTATCTTGTTGAA 3′
	Probe 5′ 6FAM-ACCCAAAGAAGAAGATGGAAAAGCGGTT-BBQ 3′
	Sense 5′ TGGCCCTGACCCAACTATGAT3′
CTGF	Antisense 5′ GCACTTTTTGCCCTTCTTAATGTT 3′
	Probe 5′ 6FAM-AGCCAACTGCCTGGTCCAGACCA-DB 3′
	Sense 5′ GGGCTTTCGCTTCAGTGCT 3′
TGF-β	Antisense 5′ TCGGTTCATGTCATGGATGGT 3′
	Probe 5′ 6FAM-TCAGTCCCAAACGTCGAGGTGACCTG-DB 3′
	Sense 5′ TGGTCCTCTGGGCATTGC 3′
Collagen-1	Antisense 5′ CACTGCCAGGGTTACCATCA 3′
	Probe 5′ 6FAM-TTCACCAGGGGCACCATTAACTCCA-DB 3′
	Sense 5′CGTGGTGAGATCTTCTTCTTCAAGGA 3′
MMP-2	Antisense 5′ CCTCATACACAGCGTAATCTTTTC 3′
	Probe 5′ 6FAM-ACACCACGTGACAAGCCCACAGGTC-DB 3′
	Sense 5′ GGAGGAAAGAAGGAATATCTAATTGCAG 3′
TIMP-2	Antisense 5′ CCAGGGCACAATAAAGTCACAGA 3′
	Probe 5′ 6FAM-CATCTTGCCATCTCCTTCCGCCTTCC-DB 3′
LOX*	Rn00566984_m1

* TaqMan™ Gene expression Assay (Applied Biosystems).

### Statistical Analysis

Results are expressed as mean ± SEM. Data was analyzed using a one-way analysis of variance, followed by Tukey or Dunnet test to assess specific differences among all groups or control animals, respectively; an unpaired Student *t* test was performed to compare scar size using GraphPad Software Inc (San Diego, CA, USA). The predetermined significance level was P<0.05.

## Results

### General Characteristics and Cardiac Hemodynamics in both Ventricles

As shown in Table 2, all infarcted animals showed a similar scar size independent of the development of HF. A reduction in body weight was observed in INF-HF as compared with the other two groups. However, the relative weights of both right and left ventricles and lung were higher in INF-HF animals than in the INF and control group (Table 2). Independent of the presence of signs of HF, HR values in INF animals were similar to those observed in controls (Table 2). Haemodynamic parameters are also shown in Table 2. The RVSP, RVEDP, +dP/dt and −dP/dt were higher in infarcted animals with HF than in those without it, which showed similar values to those of control animals. No differences were observed in arterial or LVSP among groups (Table 2). Animals with HF, however, showed higher levels of LVEDP compared to SHAM or INF groups. The inotropic indexes of contractility, +dP/dt and −dP/dt, were reduced in animals with HF as compared with the other two groups (Table 2).

**Table 2:** General characteristics.

	SHAM (n = 11)	INF (n = 15)	INF-HF (n = 8)
Body weight (g)	347±7.9	320±9.5	304±12.6 *
LV/BW (mg/g)	2.21±0.06	2.23±0.05	2.51±0.12
RV/BW (mg/g)	0.75±0.04	0.69±0.03	1.09±0.10*#
LW/BW (mg/g)	6.37±0.18	6.74±0.28	11.82±0.85*#
RV/LV+SW	0.33±0.02	0.31±0.01	0.44±0.03*#
Scar size (%)		51.7±2.3	53±1.4
SAP (mmHg)	108±4.8	103±4.1	102±5,2
DAP (mmHg)	68±3.9	71±4.7	73±5.7
Heart rate (bpm)	260±7	280±11	283±10
LVSP (mmHg)	115.2±5.17	119.5±5.38	117.1±5.15
LVEDP (mmHg)	4.11±0.4	5.17±0.7	16.12±1.99*#
LV+dP dt ^−1^ (mmHg s^−1^)	4,759±119	4,613±330	3,295±225*#
LV – dP dt ^−1^ (mmHg s^−1^)	4,230±305	4,395±199	3,224±143*#
RVSP (mmHg)	31.9±1.4	36±1.9	43±4.8*
RVEDP (mmHg)	1.7±0,17	2.7±0,44	3.7±0,33*
RV+dP dt ^−1^ (mmHg s^−1^)	1,737±96.8	1,778±123	2,607±117*#
RV – dP dt ^−1^ (mmHg s^−1^)	1,436±114	1,573±116	2,115±230*#

Body weight, relative weight of left (LV/BW) and right ventricle (LV/BW), relative weight of lungs (LW/BW), the weight ratio of right to left ventricles plus interventricular septum (RV/LV+SW), scar size, systolic (SBP) and diastolic (DBP) blood pressure, heart rate, left (LVSP)and right (RVSP) ventricle systolic pressure, left (LVEDP) and right (RVEDP) end diastolic pressure, positive (+dP/dt) and negative (-dP/dt) rates of pressure development in left (LV) and right (RV)ventricle of sham operated rats (SHAM) and infarcted animals with heart failure (INF-HF) with or without heart failure (INF) after 30 days after ligation of left coronary artery. Data are mean ± S.E.M. *p<0.05 compared to SHAM. ^#^*p*<0.05 compared to INF group.

### Collagen Content and Morphometric Analysis

**Figure 1:**
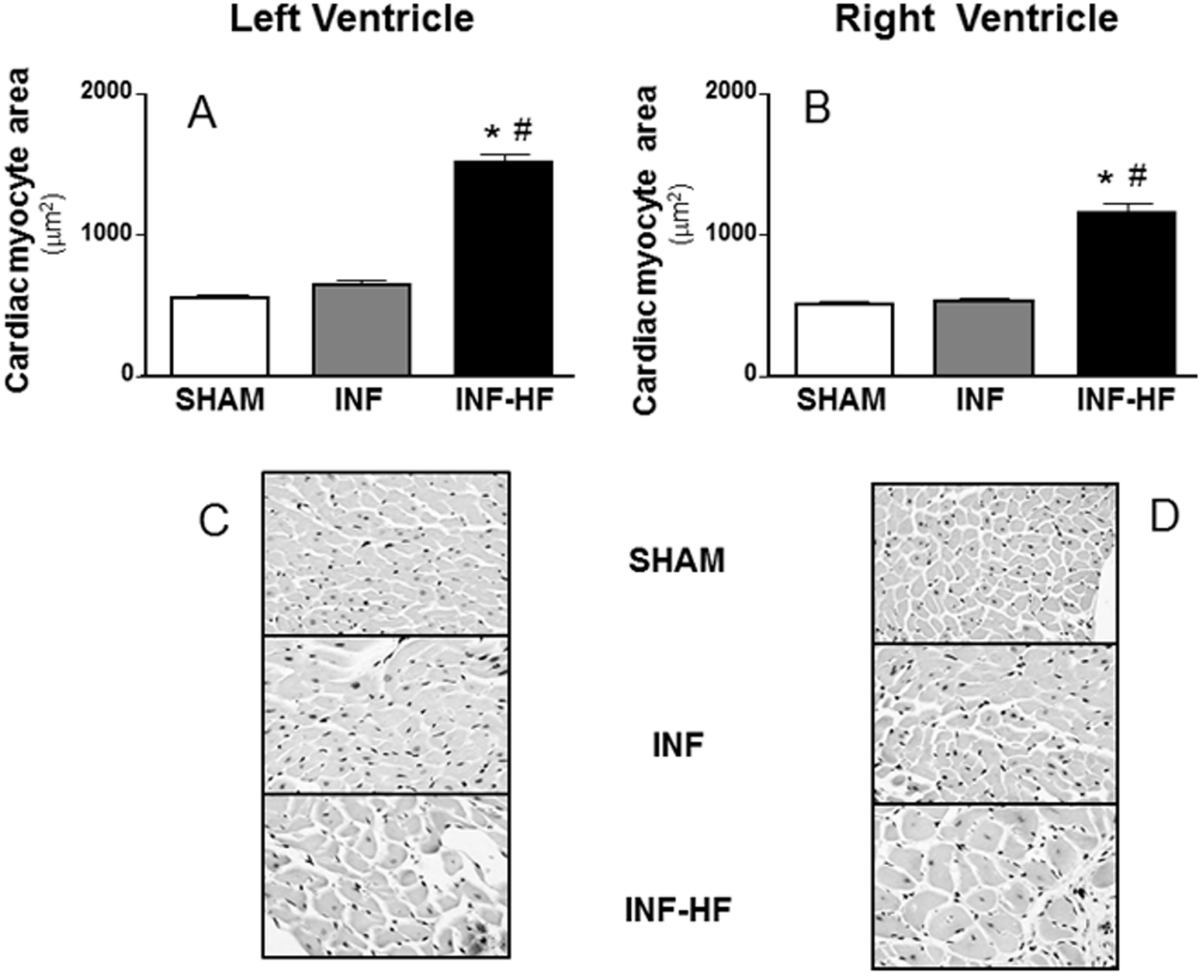
Cardiac myocyte cross-sectional area from left (A) and right ventricle (B) of sham-operated rats (SHAM) and infarcted animals with (INF-HF) or without heart failure (INF) 30 days after ligation of left coronary artery. Bottom panel (C and D) shows representative photomicrographs of hematoxilin-eosin-stained ventricular tissue sections for all groups (magnification x40). Data are mean ± S.E.M., *p<0.05 vs. SHAM group; ^#^ p<0.05 vs INF group.

**Figure 2:**
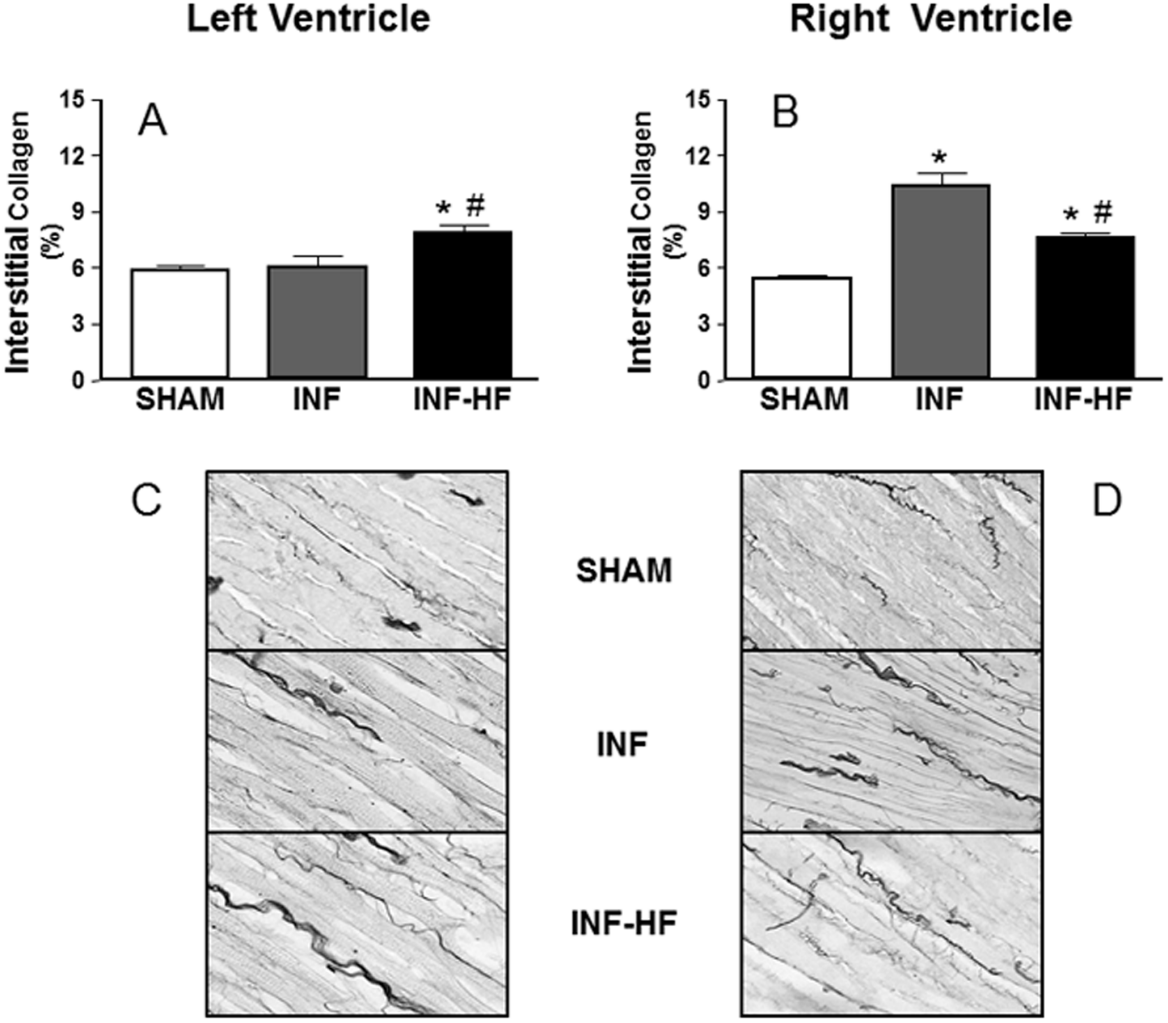
Collagen content in left (A) and right ventricle (B) of sham operated rats (SHAM) and infarcted animals with (INF-HF) or without heart failure (INF) 30 days after ligation of left coronary artery. Bottom panel (C and D) shows representative photomicrographs of Sirius red-stained ventricular tissue sections for all groups (magnification x40). Data are mean ± S.E.M. *p<0.05 vs. SHAM group; ^#^ p<0.05 vs INF group.

**Figure 3:**
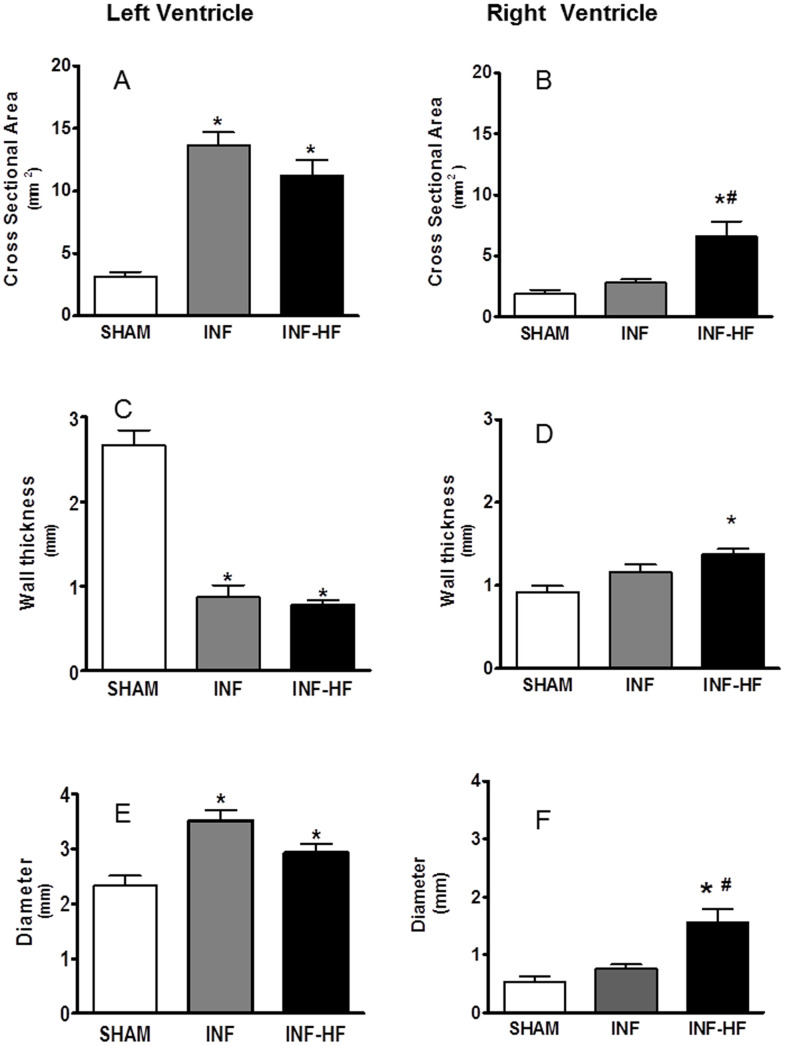
Cross sectional area and diameter of left (A and C) and right (B and D) ventricle cavities, and free wall thickness of left (E) and right (F) ventricles of sham operated rats (SHAM) and infarcted animals with (INF-HF) or without heart failure (INF) 30 days after ligation of left coronary artery. Data are mean ± S.E.M., *p<0.05 vs. SHAM group; ^#^ p<0.05 vs INF group.

Myocyte cross-sectional area was markedly increased in the INF-HF group in both left and right ventricles, but was not modified in the INF group as compared with controls (Figure 1). In the left ventricle, an increase in the interstitial myocardial collagen content was observed in the INF-HF group as compared with INF and SHAM groups (Figures 2A and 2C). In the right ventricle, INF and INF-HF animals exhibited higher interstitial myocardial collagen content than the SHAM group (Figures 2B and 2D). The accumulated area of fibrosis in the right ventricle was equally high in the INF and INF-HF groups (SHAM: 31.5±3.57 µm^2^; INF: 64.7±6.4 µm^2^; INF-HF: 54.1±7.2 µm^2^, p<0.05). As compared with control animals, all infarcted animals, independently of the presence or not of HF, show an increase in cross sectional area and diameter of left ventricle cavity, which was associated with a reduction in the thickness of its free wall (Figures 3A, 3C, 3E, respectively). However, in those that develop HF, an increase in cross sectional area, diameter of cavity and free wall thickness of right ventricle as compared with controls was also observed (Figures 3B, 3D, 3F, respectively). No differences in septum thickness were found among any group (data not shown).

### Gene Expression of Factors Involved in Fibrosis and Inflammation

**Figure 4:**
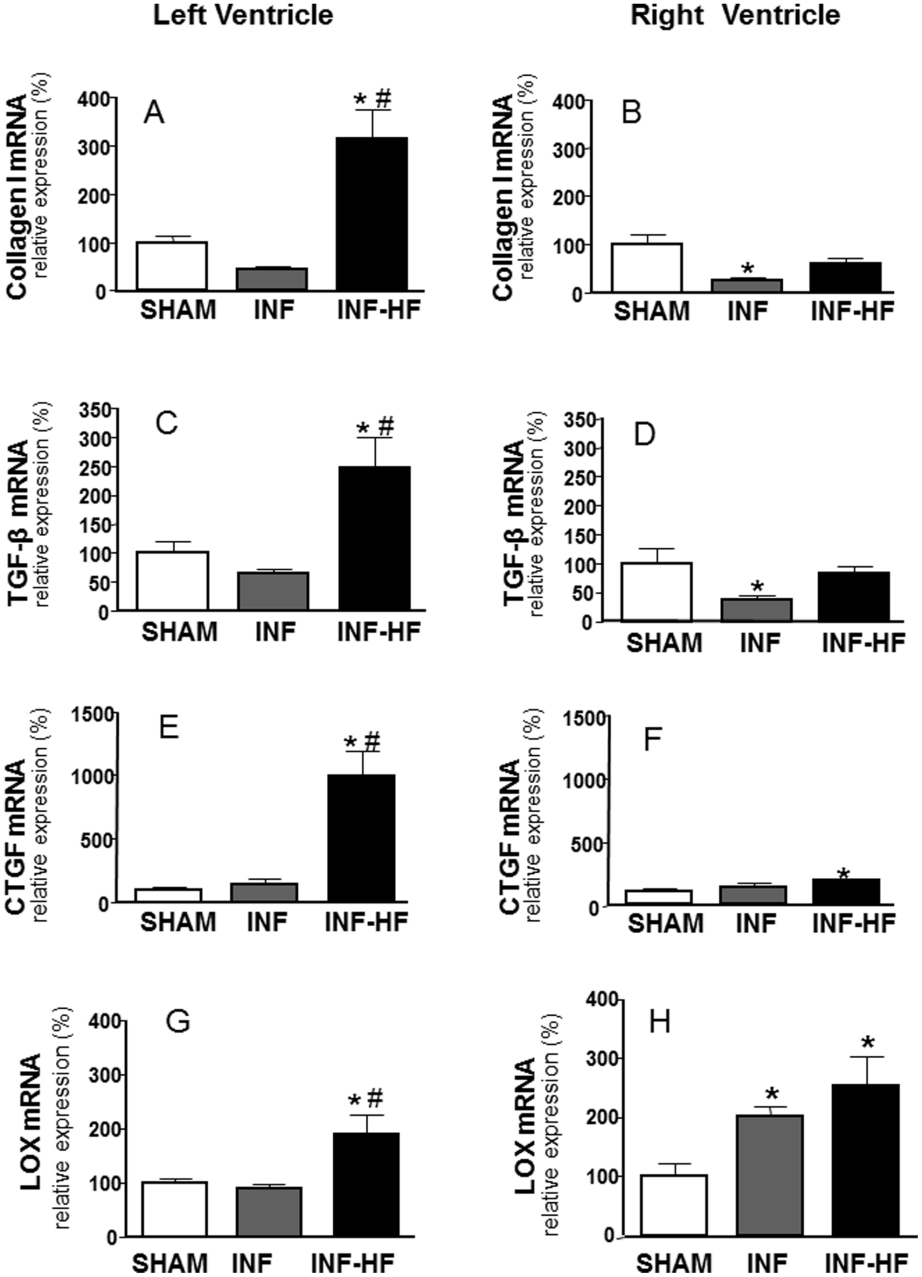
Collagen I (A and B), transforming growth factor-β (TGF-β; C and D), connective tissue growth factor (CTGF; E and F) and lysyl oxidase (LOX; G and H) mRNA levels in left (A, C, E and G) and right ventricle (B, D, F and H) of sham operated rats (SHAM) and infarcted animals with (INF-HF) or without heart failure (INF) 30 days after ligation of left coronary artery. Results were normalized by 18S expression levels. Data are mean ± S.E.M. *p<0.05 vs. SHAM group; ^#^ p<0.05 vs INF group.

**Figure 5:**
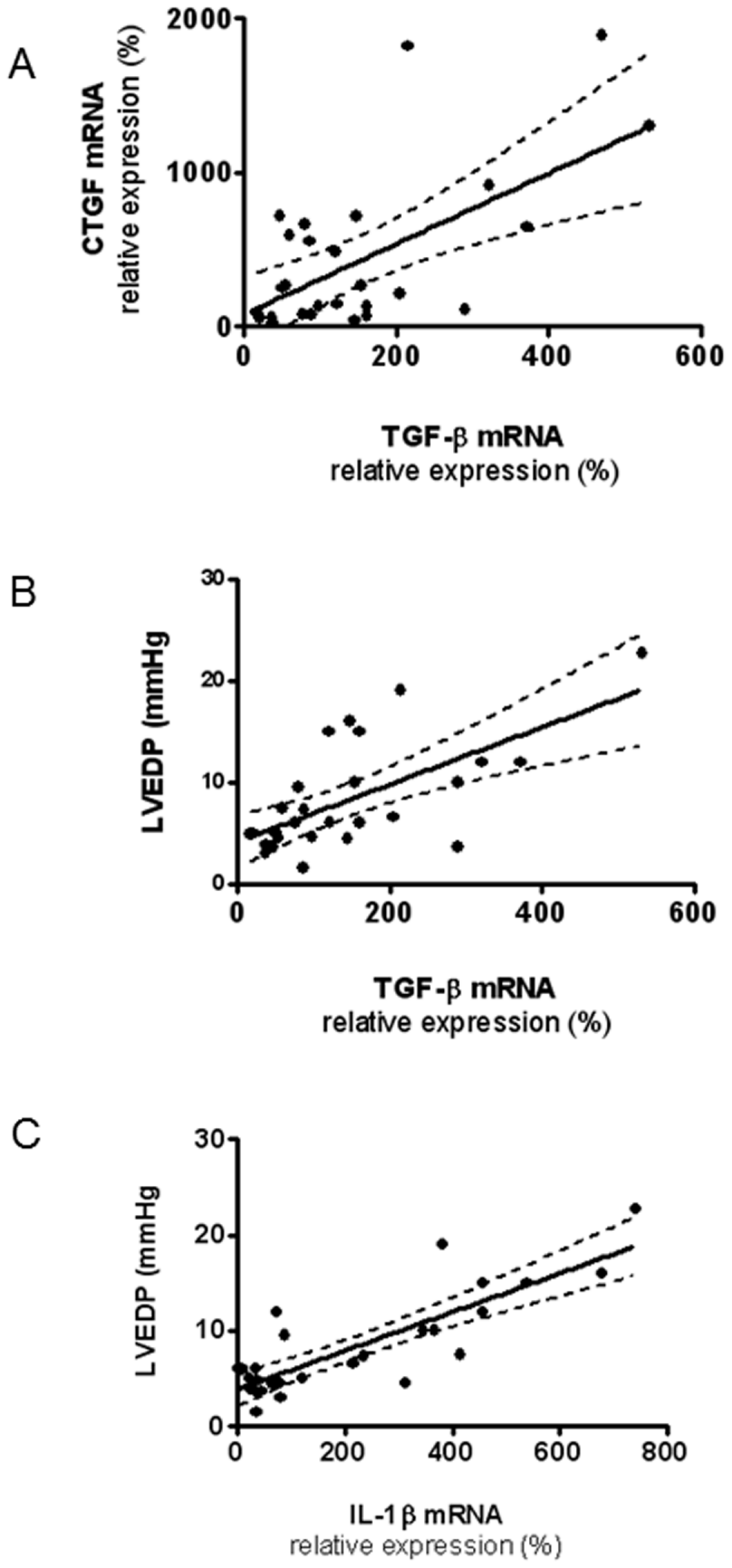
Scatter plots of the correlations between (A) mRNA levels of transforming growth factor-β (TGF-β) and connective tissue growth factor (CTGF); (B) mRNA levels of transforming growth factor-β (TGF-β) and left ventricle end diastolic pressure (LVEDP) and (C) mRNA levels of interleukin 1 beta (IL-1β) and left ventricle end diastolic pressure (LVEDP) in left ventricle of sham-operated rats (SHAM) and infarcted animals with (INF-HF) or without heart failure (INF) 30 days after ligation of left coronary artery.

**Figure 6:**
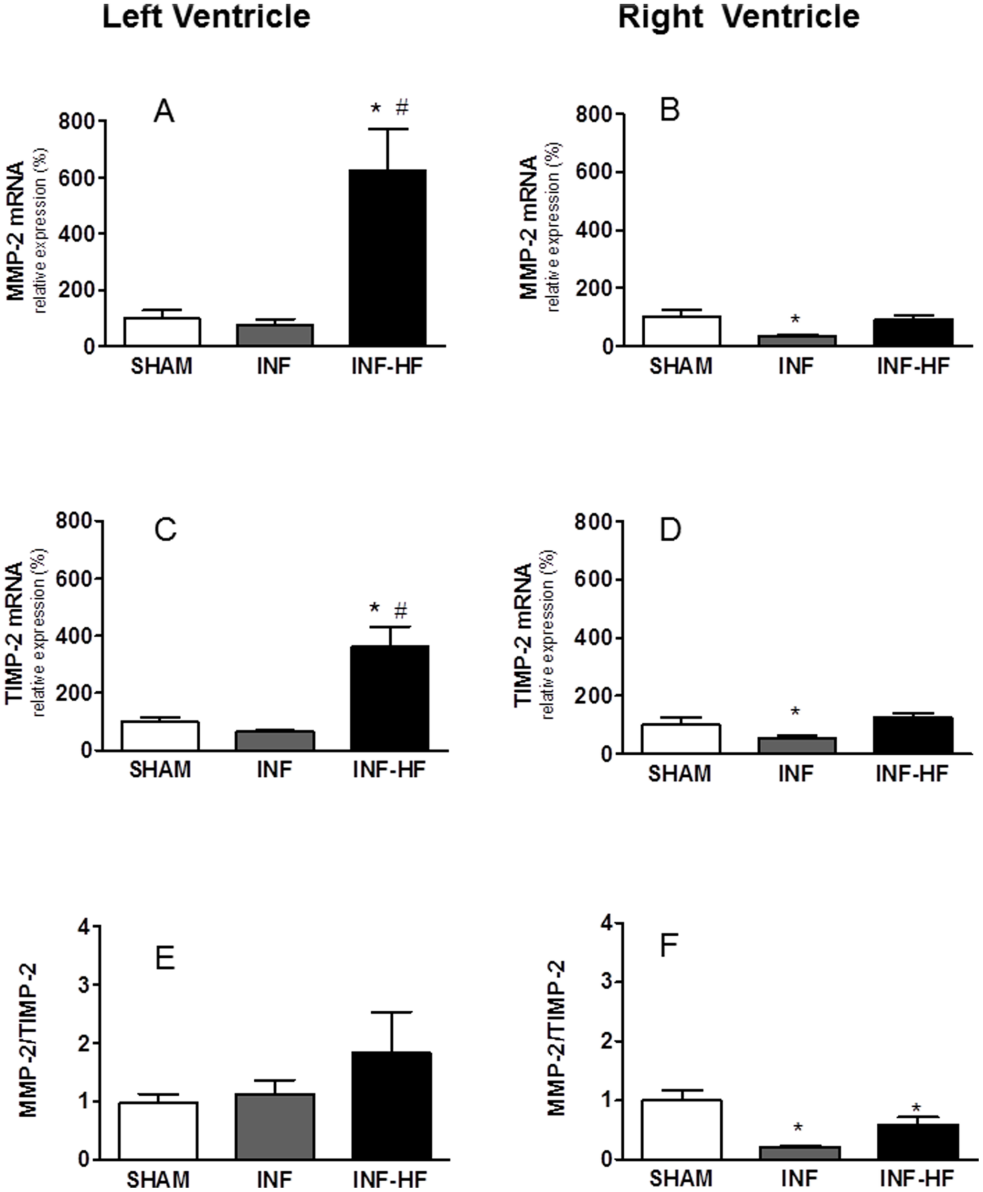
Metalloproteinase-2 (MMP-2, A and B), tissue inhibitor metalloproteinase-2 (TIMP-2, C and D) mRNA levels and MMP-2/TIMP-2 mRNA ratio (E and F) in left (A, C and E) and right ventricle (B, D and F) of sham-operated rats (SHAM) and infarcted animals with (INF-HF) or without heart failure (INF) 30 days after ligation of left coronary artery. Results were normalized by 18S expression levels. Data are mean ± S.E.M. *p<0.05 vs. SHAM group; ^#^ p<0.05 vs INF group.

We aimed to study the expression pattern of factors involved in collagen synthesis and/or degradation after MI in both ventricles in animals presenting HF or not. In fact, in the left ventricle, mRNA levels of collagen I, TGF-β, CTGF and LOX were higher in the INF-HF group than those detected in the SHAM and in the INF groups (Figures 4A, 4C, 4E, 4G, respectively). Interestingly, a significant positive correlation was found between TGF-β with CTGF (r = 0.604; p<0.007, Figure 5A) and also with LVEDP (r = 0.609; p<0.0.001, Figure 5B) in the left ventricle. Although the MMP2 and TIMP2 mRNA levels were increased in the INF-HF and it was unchanged in the INF group in the left ventricle, all animals showed a similar MMP2/TIMP2 mRNA ratio (Figure 6).

In the right ventricle of INF-HF group only the mRNA expression of CTGF and LOX were increased compared to the SHAM group (Figure 4F and 4H, respectively). On the other hand, in the right ventricle of INF animals only the expression of LOX was increased while CTGF was unchanged (Figure 4H and 4F, respectively). However the expression of both collagen I (Figure 4B) and TGF-β (Figure 4D) was reduced in the INF group and remain unchanged in the INF-HF animals. Interestingly, MMP2/TIMP2 mRNA ratio was reduced in both groups compared to SHAM (Figure 6F). This reduction was mainly due to a decrease in MMP-2 expression (Figure 6B and 6D). Taken together it seems that the main factors involved in right ventricle late remodeling are the increased LOX and the reduced MMP2 expression in both groups, while in the INF-HF group the increase of CTGF mRNA expression could also be involved.

**Figure 7:**
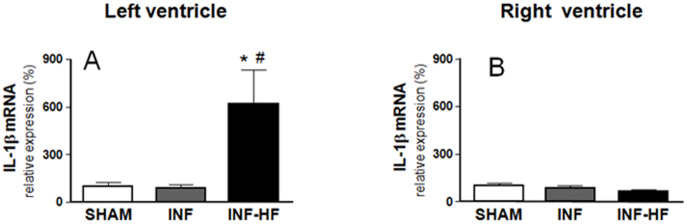
Interleukin-1β (IL-1β) mRNA levels in left (A) and right ventricle (B) of sham-operated rats (SHAM) and infarcted animals with (INF-HF) or without heart failure (INF) 30 days after ligation of left coronary artery. Results were normalized by 18S expression levels. Data are mean ± S.E.M. *p<0.05 vs. SHAM group; ^#^ p<0.05 vs INF group.

Finally, an increase in IL-1β gene expression was found in the left ventricle in the INF-HF group compared with INF and SHAM groups (Figure 7A). Furthermore, a significant correlation was found between LVEDP and IL-1β (r = 0.834; p<0.001, Figure 5C). No differences, however, were observed in IL-1β gene expression among any group in the right ventricle (Figure 7B).

## Discussion

It has been shown that wound healing after MI involves the development of a scar rich in extracellular matrix. However, collagen deposit, which contributes to adverse remodeling, also occurs in the non-ischemic myocardium of left and right ventricles, although the underlying mechanisms or mediators that can favor functional alterations are not entirely understood. Here we report that at 30 days after MI due to ligation of left coronary artery there is collagen deposit in the right and left ventricle from INF-HF animals, whereas it is present only in the right ventricle in the infarcted animals without signs of HF. We also demonstrate that mechanisms involved in the extracellular matrix deposition are still present after 30 days in the right and left ventricles and appear to be different in each one. While, in the right ventricle of both groups seems to be a consequence of reduction in the extracellular matrix degradation, an increase in collagen production, however, was the main mechanism involved in the left ventricle of the INF-HF group. In addition, only infarcted animals that developed HF showed myocyte hypertrophy in both ventricles.

Animals with HF showed increased contractility and relaxation index (dP/dt+and −, respectively) in the right ventricle, which could be partially due to increased RVEDP leading to a higher RVSP as described by Frank-Starling mechanism of the heart [22]. These functional alterations could be a response to the increased afterload in the right ventricle due to augmented LVEDP observed in these animals, and which could have induced an increase in the pulmonary arterial resistance [23]. This fact could explain not only the hypertrophy and the dilatation observed in the right ventricle in the INF-HF group but also the free wall thickening found in this group. These changes do not depend on the left ventricle scar size or the presence of fibrosis in the non-infarcted myocardial of the right ventricle as it was not different between INF and INF-HF groups. It seems that right ventricle function can be reduced after MI in absence of pulmonary hypertension since a reduction in ejection fraction has been observed in mouse with coronary artery ligation [24] and in patients with acute myocardial infarction [25]. Coronary artery ligation was accompanied by left ventricular dilatation suggesting eccentric remodeling, which is a common consequence of MI [26]–[28]. This process results from side-to-side slippage of the cardiomyocytes in the surviving myocardium [26] and could account for most wall thinning observed in these animals. Dilatation of the ventricle can initially play a compensatory role as described by Frank-Starling mechanism of the heart [22], but maintained over time it can trigger the development of HF [22]. By contrast, although the INF group presented the same left ventricle scar area and ventricular dilatation, neither functional nor hypertrophy alterations were found in this chamber. In the left ventricle of HF animals, ventricular dilatation and wall thinning suggest an increase in wall tension [26] that could favour the cardiac hypertrophy and fibrosis in these animals and that can result in greater degree of cardiac dysfunction. In the INF-HF group, the increase in extracellular matrix could explain the reduced contractility and relaxation observed in these animals. These results are in agreement with previous studies that show that collagen deposit, mainly in the interstitium of the uninfarcted remote left ventricle, causes ventricular stiffness and mechanical dysfunction that contributes to the evolution of HF by favoring geometric changes [8], [29]. Interestingly, there was observed in the INF group an increment in collagen deposit only in the right ventricle, even though LVEDP was normal. Some studies have observed that patients with cardiac remodeling after acute MI had a significant increase in apoptosis in right ventricle, even when it is spared from initial ischemic damage [30], [31]. Therefore, collagen accumulation observed in the right ventricle in infarcted animals can be considered to be a reparative process designed to replace damaged and lost cardiac myocytes. However, the precise mechanisms regarding how MI of the left ventricle can affect remodeling of the right ventricle seems not yet to be clear [31].

It has been demonstrated that the remodeling process in the ventricles begins soon after the ischemic injury and continue over months [7], [8]. So the understanding of late mechanisms involved in the remodeling process is also important to elucidate the physiopathology of heart failure bringing therapeutic possibilities. We demonstrated that left ventricle late remodeling process, associated to fibrosis, seems to be present in the HF animals, considering the increase in collagen I gene expression. Similarly, an increase not only in gene expression of collagen I but also in that of collagen III and collagen VI have been reported in left ventricle in different models of MI only a few days after coronary artery ligation [32], [33]. These animals also expressed higher LOX levels in left ventricle, the enzyme responsible for collagen assembly that makes it less susceptible to proteolytic degradation [13], [14]. The up-regulation of LOX expression seems to be an early event after MI since Tsuda et al [33] have reported high mRNA levels in both ischemic and in remote non-ischemic areas of left ventricle in mouse 3 days after distal left coronary artery ligation. This up-regulation correlates with collagen deposition and scar formation in the infarcted area. A similar increase in LOX has been found in left ventricle of patients with myocardial fibrosis and dilated cardiomyopathy [34] or HF [14]. Because an increase in collagen cross-links enhances ventricular stiffness and reduces compliance, LOX up-regulation could compromise ventricular function in cardiac diseases [14], [15] and could underlie the alteration of left ventricle relaxation and contraction observed in our study. In addition, this supports the idea that a minor degradation could also be involved in the fibrosis observed in INF-HF, even considering that all groups show a similar MMP-2/TIMP-2 ratio. Interestingly, in the right ventricle of both INF and INF-HF animals, a late remodeling process seems to be also present, since it was observed a reduction in both MMP-2/TIMP-2 ratio (mainly due to a reduction in MMP-2 expression) and collagen I mRNA, as well as an increase in LOX expression. These findings suggest a reduction in collagen degradation rather than increase in its production.

Our results demonstrated an increased expression of TGF-β and CTGF mRNA (associated with interstitial fibrosis) in the left ventricle from INF-HF animals. TGF-β and CTGF have been considered to be mediators of collagen production in the heart. In fact, these growth factors promote extracellular matrix synthesis and mediate cardiac fibrosis associated with MI and other cardiac diseases [9], [32], [35]–[37]. In addition, they could also be involved in LOX production since TGF-β and CTGF are some of the factors involved in the up-regulation of LOX in different settings [13], [14], [38], [39], and strategies aiming to block TGF-β biological activity reduced abnormal LOX expression and collagen crosslinking in the heart [13]. The positive correlation between TGF-β and CTGF gene expression could be explained by previous results demonstrating that a signalling pathway, TGF-β/Smad, may lead to CTGF up-regulation and subsequent cardiac fibrosis [20], [35]. Besides its role in the stimulation of extracellular matrix production, high TGF-β levels observed in animals with HF can exert additional actions involved in ventricular remodeling, such as conversion of fibroblasts to myofibroblasts, inhibition of MMPs and proinflammatory actions, as has been reported in different studies [10]. All these data therefore support a major role of this cytokine in the changes that occur after MI, which can be involved in the development of HF. Besides exerting a profibrotic effect, CTGF induces cardiac myocyte hypertrophy [40]. The fact that animals with HF show cardiac myocyte hypertrophy in both ventricles accompanied by an increase in CTGF mRNA levels supports a role of CTGF in the cardiac myocyte hypertrophy observed in this group. In fact, in the left ventricle of the INF group where interstitial collagen and cardiac myocyte area did not change, the expression of TGF-β and CTGF mRNA was not modulated.

As already reported [8], [41], an inflammatory process was observed in left ventricle of those animals that developed HF after MI, as suggested by the upregulation of IL-1β. It has been shown that an inflammatory process is an early response after MI, which can contribute to the proteolytic digestion and phagocytosis of the damaged tissue. In addition, cytokines can contribute to cardiac function alterations by participating in post–infarction remodeling [42], [43]. This potential role seems to be a consequence of the ability of IL-1β to modulate MMP activity (specifically, MMP-2 and MMP-9), as has been demonstrated in *in vivo* and *in vitro* studies [44]. Along these lines, we have observed an increase in MMP-2 gene expression in left ventricle in those animals with HF, and in which IL-1β mRNA was also elevated. However, these animals also showed a parallel increase in TIMP-2, and the ratio MMP-2/TIMP-2 was therefore unchanged. Since these factors are related to collagen synthesis and degradation it could be related to the late remodeling process observed in the left ventricle of INF-HF animals. A similar situation has been reported for MMP-1 and its inhibitor TIMP-1 in rats 4 weeks after MI (increases in both MMP-1 and TIMP-1 gene and protein levels) [41].

In summary, our results demonstrated that 30 days after MI animals presenting signs of HF or not showed a different pattern of remodeling in both chambers independent of scar size. MI produced LV dilatation and free wall thinning. The eccentric remodeling was also accompanied by fibrosis in the HF group, The mechanisms involved in the LV fibrosis that starts soon after ischemic injury are still present after 30 days since was found an increase in collagen production, as suggested by the increase in gene expression of collagen as well of the profibrotic factors CTGF and TGF-β. On the other hand, RV from HF animals presented dilatation, while fibrosis and reduction in collagen degradation occurred in the RV of both groups. One important limitation of this study is the fact that an analysis of MMPs, TIMPs, collagen isoforms, and cytokines gene expression and MMP activity or expression still necessary, in both chambers, in order to reinforce the present hypothesis.

The data strengthens the complexity and clinical relevance of ventricular remodeling after MI because even if the fibroses ad scar area are already well defined in the ventricle, signaling pathways are still activated after 30 days and seems to be different in each chamber. Therefore, the understanding of molecular events occurring at the surviving area after MI is important to a better management of patients after coronary occlusion.
